# Imaging flow cytometry: from high - resolution morphological imaging to innovation in high - throughput multidimensional biomedical analysis

**DOI:** 10.3389/fbioe.2025.1580749

**Published:** 2025-05-09

**Authors:** Qing Huang, Zhengyu Zhou, Qiao Lv, Qian Min, Lu Jiang, Qian Chen, Jin Peng, Hongli Zhou, Ju Zhou, Qian Dai, Jianyun Zhou

**Affiliations:** ^1^ Clinical Medical Research Center, Xinqiao Hospital, Army Medical University, Chongqing, China; ^2^ Department of Epidemic Prevention, 92914 Hospital, Hainan, China

**Keywords:** imaging flow cytometry, high - throughput analysis, morphological imaging, biomedical research, artificial intelligence, machine learning

## Abstract

Imaging flow cytometry (IFC), as an extension of conventional flow cytometry, has emerged as a cutting-edge cellular analysis tool by integrating high-resolution imaging technology, and has shown significant potential and application value in biomedical research. In this paper, we comprehensively review the evolution of IFC from its early theoretical development to its current mature application, and explain its working principle, unique advantages, and the current status of its application in several biomedical fields. The paper focuses on how IFC integrates high-throughput and morphological imaging, highlighting its key role in cell biology, immunology, oncology, and environmental monitoring. Furthermore, the paper addresses the challenges and opportunities in data analysis, and proposes the potential of artificial intelligence (AI) and machine learning technologies to drive its progress. The paper concludes with an outlook on the future of IFC, predicting its application in emerging research areas and emphasizing the role of continuous technological innovation in driving the development of the field. It aims to provide researchers with a comprehensive view of IFC to promote its widespread application in biomedical research.

## 1 Introduction

Flow Cytometry (FC) is a revolutionary biotechnology that allows scientists to make rapid, simultaneous measurements of a wide range of physical and chemical properties of cells (Lazarski and Hanley, 2024; Railean and Buszewski, 2022). Since its origin in the 1950s, FC has undergone significant technological advances and its applications have expanded from the initial counting and size analysis of cells to the multiparametric analysis of cellular functions (Cram et al., 1992; Zhao et al., 2023). The basic principle of FC involves suspending cells or particles in a fluid and passing them one by one through an extremely narrow detection channel. When cells move through the laser beam, signals generated by their physical properties (e.g., forward and side scattered light) and chemical properties (e.g., fluorescent labeling) are captured by the detector and transformed into electrical data, which computers then process to produce statistical information and graphical presentations about the cell properties (Robinson and Roederer, 2015; Zorzi et al., 2023).

Technological advances have driven the multi-stage evolution of FC: Initially, the creation of multicolor FC enabled the parallel analysis of multiple parameters using multiple fluorescence channels simultaneously, which greatly improved the efficiency in studying cellular properties (De Rosa et al., 2003). This was followed by adding fluorescence-activated cell sorting (FACS) techniques that not only added the ability to analyze, but also provided the ability to physically separate specific cell populations, which has been of great significance for in-depth research and experimentation (Fulwyler, 1965). The advent of spectral flow cytometry introduced a wider spectral range and upgraded optics, which greatly improved the resolution and sensitivity of fluorescence detection (Brandi et al., 2023; Sharma et al., 2024). The introduction of mass spectrometry flow cytometry is a perfect combination of FC and mass spectrometry techniques, which employ heavy metal isotopes as labels. This integration facilitates the concurrent analysis of over 40 parameters on a per-cell basis, and effectively circumvents the problem of overlapping fluorescence signals (Spitzer and Nolan, 2016). Perhaps most notable is Imaging flow cytometry (IFC), which incorporates high-resolution imaging techniques capable of analyzing the physical and chemical characteristics of cells while capturing morphological images of cells, providing intuitive information on cellular function and allowing researchers to gain insight into morphological changes and microstructure in a high-throughput environment (Rees et al., 2022).

The origin of IFC was driven by the need for deeper cellular analysis. Although conventional FC enables high-speed, multi-parameter cell detection and analysis, it lacks the ability to visualize cell morphology and microstructure. To break through this limitation, researchers have begun to explore new ways to combine imaging technology with FC. Thanks to the rapid development of imaging technology, especially the breakthroughs in digital imaging and high-speed camera technology, a solid technical foundation has been laid for the realization of IFC (Han et al., 2016). In addition, the increase in computer processing speed and the innovation of data analysis algorithms have made it possible to rapidly process large amounts of complex data, which creates the conditions for the application of IFC. The concept of IFC emerged, which aims to integrate the advantages of high-throughput analysis of conventional FC and the morphological details of microscope imaging technology (Stavrakis et al., 2019).

The birth of IFC is the result of the cross-fertilization of several disciplines, including biology, optics, and engineering. This interdisciplinary cooperation model provides a broader perspective and richer resources for its development, and accelerates its translation process from theory to practice. Since the debut of the first commercial IFC system, the Amnis ImageStream100 (Luminex Corporation) in 2005, the field has witnessed substantial growth, with multiple manufacturers introducing sophisticated platforms to meet diverse research needs. Today, the IFC landscape comprises a range of cutting-edge instruments, such as the Thermo Fisher Scientific Attune CytPix, which leverages acoustic focusing for high-speed morphological imaging, and the BD FACSDiscover™ S8, equipped with focusless imaging technology to enable real-time cellular visualization during high-throughput analysis. At present, the development of IFC has made a significant leap forward.

IFC is an advanced biotechnology that blends conventional FC with high-resolution imaging. By capturing high-resolution images of cells as they pass through the detector, IFC provides morphological information including cell size, shape, intracellular granularity (e.g., size and distribution of cytoplasmic or nuclear particles), and finer structural features (e.g., membrane contours, subcellular organelle morphology). The value of this technology is mainly reflected in the following aspects: 1) Morpho-functional integration: Unlike conventional FC, which lacks detailed morphological analysis, IFC can provide both morphological images and functional parameters of the cells such as cell size, shape and fluorescent labeling simultaneously, providing a more comprehensive perspective for cell analysis. 2) Visual intuition for cell classification: IFC’s imaging capability enables direct visualization of cell morphology, facilitating rapid identification of cell types and detection of abnormal features, whereas FC relies solely on fluorescent labeling and scatter signals, which may miss subtle morphological cues. 3) High-throughput precision: While inheriting the quantitative and qualitative capabilities of FC for high throughput (capturing thousands of cells per second), IFC enhances analytical accuracy by incorporating morphological metrics, reducing reliance on subjective manual gating and improving the reliability of rare cell detection. 4) Enabling new research frontiers: IFC addresses gaps in FC by facilitating studies of cell-cell interactions and subcellular dynamics, which require spatiotemporal morphological data unattainable with conventional FC. 5) Automated, objective analysis: Advanced software in IFC automates image processing and multi-dimensional data integration, minimizing human bias—an advantage over FC’s more manual, gating-dependent workflows, particularly for complex datasets. In conclusion, the advent of IFC signifies the advancement of cell analysis technology to a higher dimension and deeper level. It is projected to become more crucial in the realm of scientific research in the future.

IFC, as an emerging technology, bridges the gap between conventional FC and microscopic imaging. However, there is a relative paucity of comprehensive reviews of its technical development, application cases, and future potential, which limits the full understanding of the potential of the technique by both researchers and clinical specialists. Therefore, this paper reviews the development of IFC from its early conceptualization to the mature application of modern technology. The technical principles of IFC are described, including its links and differences with conventional FC. Describe the applications of IFC in different biomedical research fields, such as cell biology, immunology, oncology and environmental monitoring, to demonstrate its technical advantages and practical value. Explore the challenges IFC faces in data analysis and how artificial intelligence (AI) and machine learning technologies can facilitate its development. Finally, the future direction of IFC is envisioned, predicting its potential application in emerging research areas. This paper’s exploration and discourse aim to offer researchers a comprehensive perspective on IFC and promote its wider application in biomedical research.

## 2 Technical principles of IFC

**FIGURE 1:**
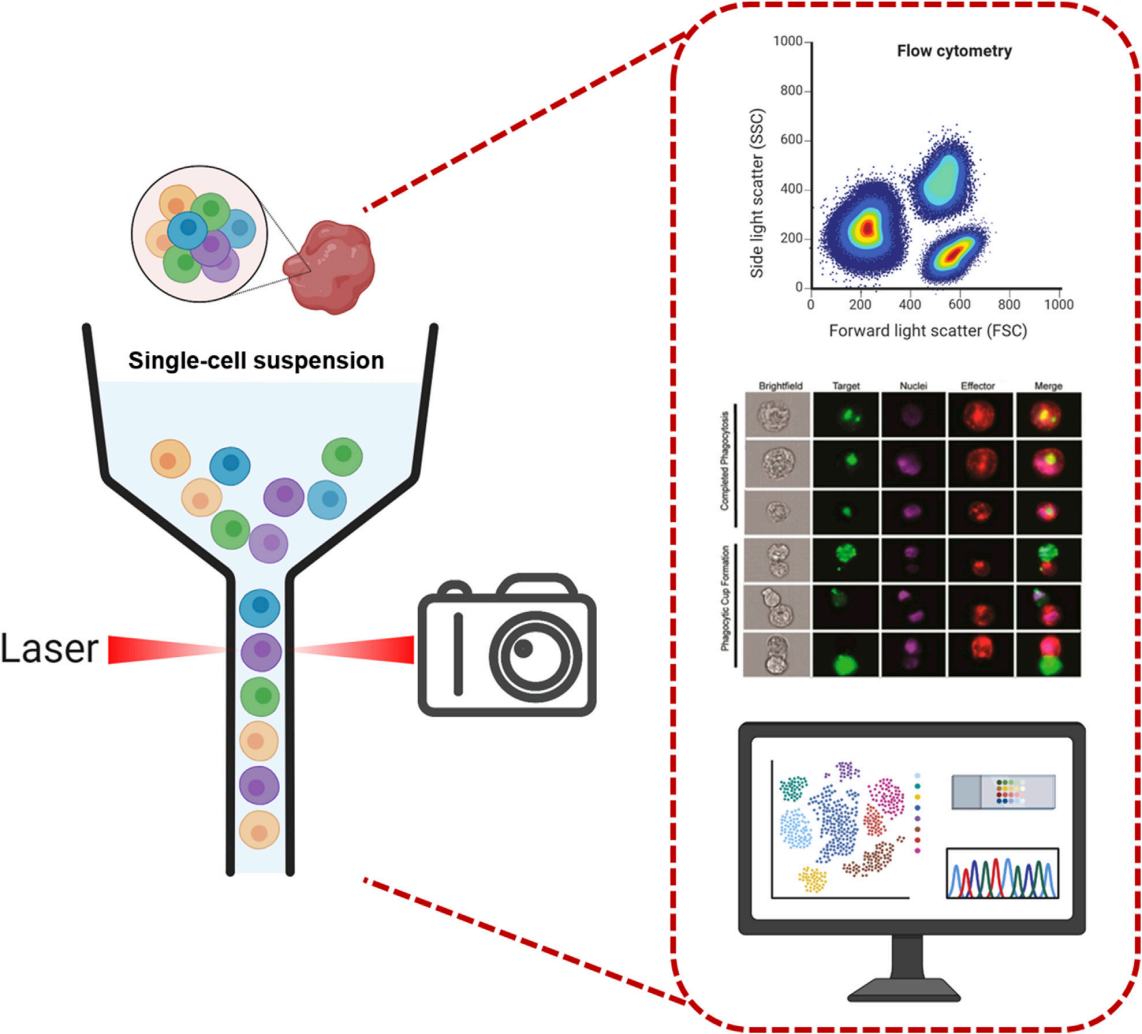
Imaging flow cytometry system diagram. The diagram illustrates the core components of an IFC system, including: 1) Fluid system: Microfluidic channels and sheath fluid mechanisms that align cells into a single-file stream for stable flow through the detection zone. 2) Optical system: Laser sources and optical filters that generate and isolate excitation/emission signals from fluorescently labeled cells. 3) Imaging system: Capture high-resolution cellular images. 4) Electronic systems: Signal processing units that convert optical signals to electrical data for downstream analysis. This integrated design enables simultaneous multi-parameter analysis and morphological imaging at the single-cell level, bridging the capabilities of conventional flow cytometry and microscopy (McEnaney et al., 2014). Copyright ^©^ American Chemical Society. Scheme images were partly created with Biorender.com.

IFC is an advanced bioanalytical instrument that combines the advantages of conventional FC and imaging technology, and is capable of obtaining high-resolution images of each cell while performing multi-parameter analysis at the single-cell resolution. The basic structure consists of (Figure 1) 1) Fluid system: the fluid system of an IFC is responsible for moving the cell sample through the instrument at a suitable rate and stability. This typically involves the use of a series of microfluidic channels and sheaths, the latter of which are used to maintain the stability of the cells during flow and to ensure that the cells move through the detector one by one in a smooth manner. 2) Optical system: This consists of a laser and optical filters used to irradiate the sample and to generate scattered light and fluorescence signals from the cells. The choice and configuration of the laser source have a direct impact on signal quality and intensity, while optical filters are used to select specific wavelengths of light to capture specific fluorescent markers. 3) Imaging system: one of the core components of an IFC, which usually includes a high-precision camera (such as a charge-coupled device (CCD) camera) and an objective lens. Alternatively, it may employ fluorescence imaging via radiofrequency-tagged emission (FIRE), which uses the beating of a digitally synthesized light field to map the image into the radiofrequency spectrum for imaging (Diebold et al., 2013). As cells pass through the detection area, the imaging system captures high-resolution images of the cells, which can be used for subsequent morphological analysis. 4) Electronic systems: include electronic devices for signal processing and data acquisition. These electronic systems are responsible for converting optical signals into electrical signals, which are further processed into analyzable data and ultimately stored for subsequent analysis.

The general workflow of IFC begins with cell preparation and labeling, where cell samples are precisely fluorescently labeled, allowing the organelle or protein of interest to be stained and emit a unique fluorescent signal for identification and analysis. This is followed by a cell flow and focusing phase, where labeled cells are introduced into the fluidic system and formed into a precisely aligned row by the sheath fluid, ensuring that each cell passes uniformly and individually through the detection zone and is exposed to the light source. As the cells pass through the detection zone, the excitation and signal capture session is initiated. The laser light source illuminates the cells and excites the fluorescent dyes inside the cells to emit fluorescence at different wavelengths; at the same time, the presence of the cells alters the scattering pattern of the illuminated light. These fluorescent and scattered lights are then collected by the signal conversion and imaging system. The fluorescent signals are converted to electrical signals and sorted by wavelength, while the imaging system captures high-resolution images that demonstrate cell morphology, size, and other visual features.

The integration of imaging technology and FC faces several technical difficulties in the development of IFC (Basiji and O'Gorman, 2015; Doan et al., 2015). The first problem is the requirement for rapid imaging and data processing capabilities. Due to the fast speed of cells passing through the detection area, the imaging system must have the ability to capture images quickly and simultaneously process and analyze the large amount of image data generated instantly, which undoubtedly puts forward higher requirements for data processing algorithms and hardware performance. Secondly, preserving the high clarity and resolution of images amidst rapid cell flow poses a challenge, ensuring that the image will not be blurred due to the rapid movement of the cells. Coupled with the complexity inherent in multiparametric imaging, which involves the simultaneous detection of multiple fluorescent markers, the problems of spectral overlap and signal crosstalk must be overcome, which further increases the complexity of the instrumentation as well as the precise requirements of the optical system. Finally, the integration of high-resolution imaging systems with high-speed data processing capabilities also results in imaging flow cytometers that are costly and complicated to maintain and operate, which somewhat limits their widespread use in laboratories and healthcare organizations with limited budgets.

To cope with these technical difficulties, a suite of innovative strategies can be implemented to facilitate the advancement of IFC. In terms of imaging and data processing, the use of high-speed complementary metal-oxide-semiconductor (CMOS) or charge-coupled device image sensor (CCD) high-speed cameras can realize rapid imaging and capture cells in a high-speed flow. At the same time, the utilization of graphics processing unit (GPU) acceleration and parallel data processing techniques can significantly increase the data processing speed and approach the goal of real-time data analysis. To maintain image clarity and resolution, focusless imaging techniques that are not limited by flow control requirements can also be introduced, such as the FIRE technology (Diebold et al., 2013; Schraivogel et al., 2022). Optimizing the microfluidic channel design and adjusting the cell flow rate, combined with increasing the camera shutter speed, effectively reduces the movement of cells in the imaging area and results in clearer images. In addition, the development of new technologies such as time delay integration (TDI) camera technology reduces motion blur and enables clear imaging under high-speed flow. To balance high-throughput analysis with the requirements for high-resolution imaging and reliable machine learning-artificial intelligence (ML-AI) algorithms data, most commercial IFC systems operate within a flow rate range of ∼10,000 images/second. This range is carefully optimized to ensure single-cell alignment and minimize motion blur, which is essential for accurate morphological and functional analysis, and thus, reliable data for ML-AI algorithms. For multiparameter imaging, the use of advanced optical filters with high spectral resolution and multipoint excitation sources helps to achieve better spectral separation between different fluorescent markers, reduce signal crosstalk, and improve the accuracy of multiparameter imaging. In response to the need to process large amounts of data, the establishment of cloud-based data processing and storage services not only reduces the dependence on local high-performance computing resources, but also enables remote access and sharing of data. Finally, the development of open-source software (such as CellProfiler) and standardized operational processes can reduce overall costs and improve accessibility and operational consistency among different users. Together, these measures can advance the development of IFC technology and enable it to be better used in laboratories and healthcare organizations with limited budgets, further facilitating the development of biomedical research.

## 3 Application of IFC

### 3.1 Cell biology and cell signaling

IFC has emerged as a significant research tool in cell biology and cell signaling. It can not only quantitatively evaluate cell surface and intracellular markers, realize the precise classification of cell subpopulations, but also reveal the localization information of proteins. In addition, IFC excels in analyzing cell cycle, apoptosis and intracellular signaling. It greatly facilitates the understanding of cell behavior and signaling mechanisms by providing detailed morphological and functional information about cells.

**FIGURE 2:**
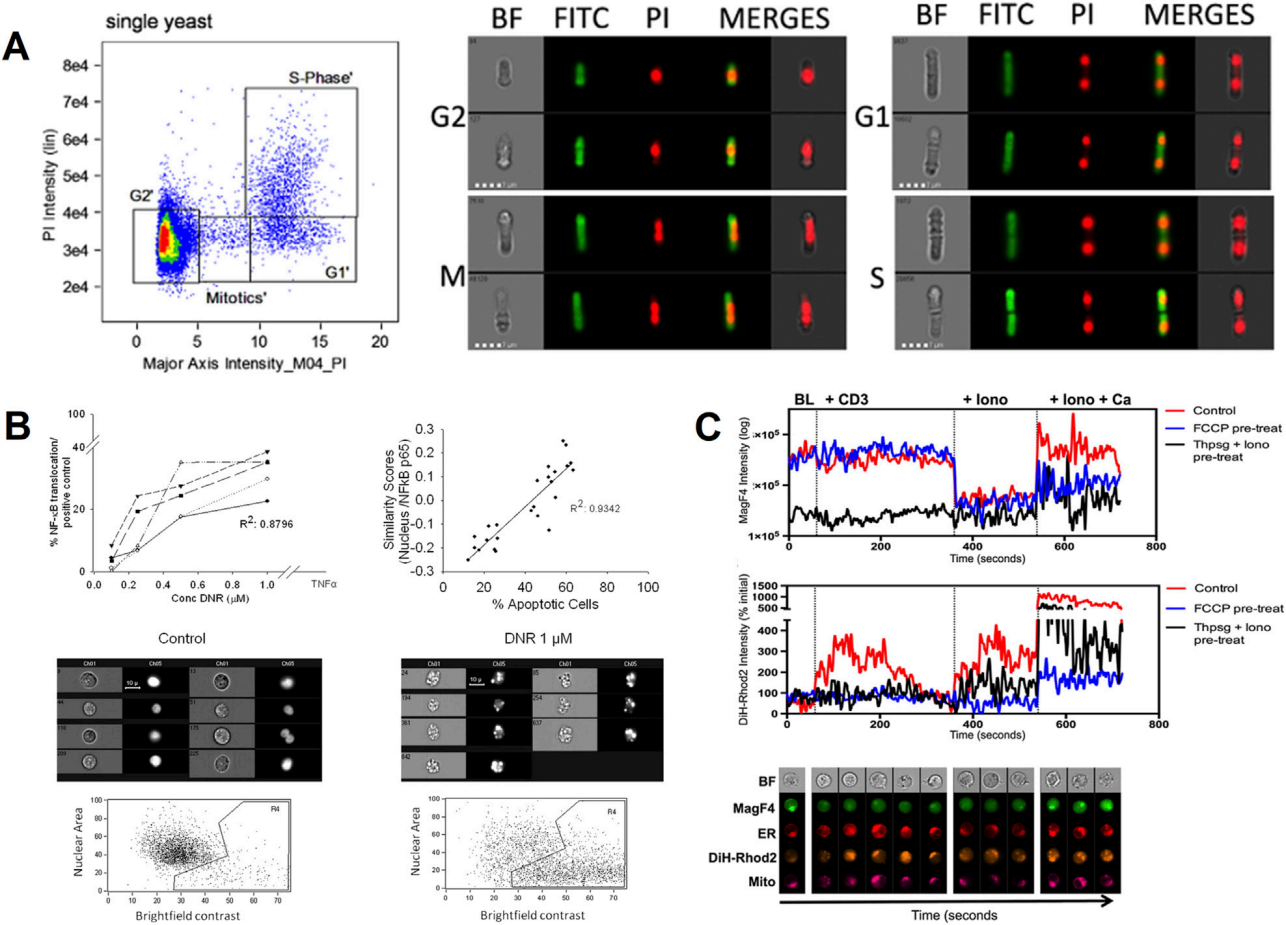
The IFC applications in cell biology and cell signaling. **(A)** Cell cycle analysis in fission yeast: The 2D plot shows propidium iodide (PI) emission intensity against signal length (major axis intensity) to distinguish G1, S, and G2/M phases. Exemplary brightfield (BF) and fluorescent images (middle and right panels) visualize DNA content changes during the cell cycle (Patterson et al., 2015). Copyright ^©^ Elsevier Ltd. **(B)** NF-κB activation monitoring: The HL-60 cell line was treated with an activator of NF-κB. The correlation between NF-κB/p65 nuclear translocations and biological responses was evaluated by IFC (Maguire et al., 2011). Copyright ^©^ Wiley-VCH. **(C)** Mitochondrial (Mito) and endoplasmic reticulum (ER) Ca^2+^ dynamics: IFC’s multispectral capabilities assess spatiotemporal changes in endoplasmic reticulum and mitochondrial Ca^2+^ signaling in T cells under stimulation, revealing organelle interactions during cell activation. The cell imaging panels at the bottom represent exemplary images of cells at each time point (Cerveira et al., 2015). Mag-Fluo4-AM (MagF4, A fluorescent marker), Dihydro-Rhod2 (DiH-Rhod2: A fluorescent dye specifically targeting the endoplasmic reticulum). Copyright ^©^ Elsevier Ltd.

Patterson, J. O. and other researchers used IFC to analyze the fission yeast cell cycle after fixation (Figure 2A). They accurately determined the G1, S, and G2/M stages of the cell cycle by monitoring the changes in DNA content and ascertained the distribution of cells across these phases using quantitative analysis (Patterson et al., 2015). George, T. C. and other investigators used IFC to observe morphological changes such as cell shrinkage, nuclear condensation, and DNA breaks during apoptosis and to precisely differentiate different modes of cell death by fluorescent labeling technique (George et al., 2004). Maguire, O. et al. used this technique to quantify the expression of p65 during NF-κB activation in the nucleus and correlated it with the results obtained by the western blot technique to assess the activation status of cellular signaling pathways (Figure 2B) (Maguire et al., 2011). Cerveira, J. et al. used IFC to study changes in intracellular structures such as the reorganization of the endoplasmic reticulum, mitochondria and cytoskeleton, which are closely related to signaling. They achieved a high-precision study for high-throughput analysis of the spatiotemporal dynamics of calcium ion signaling in T cells under various stimuli (Figure 2C) (Cerveira et al., 2015).

### 3.2 Immunology

**FIGURE 3:**
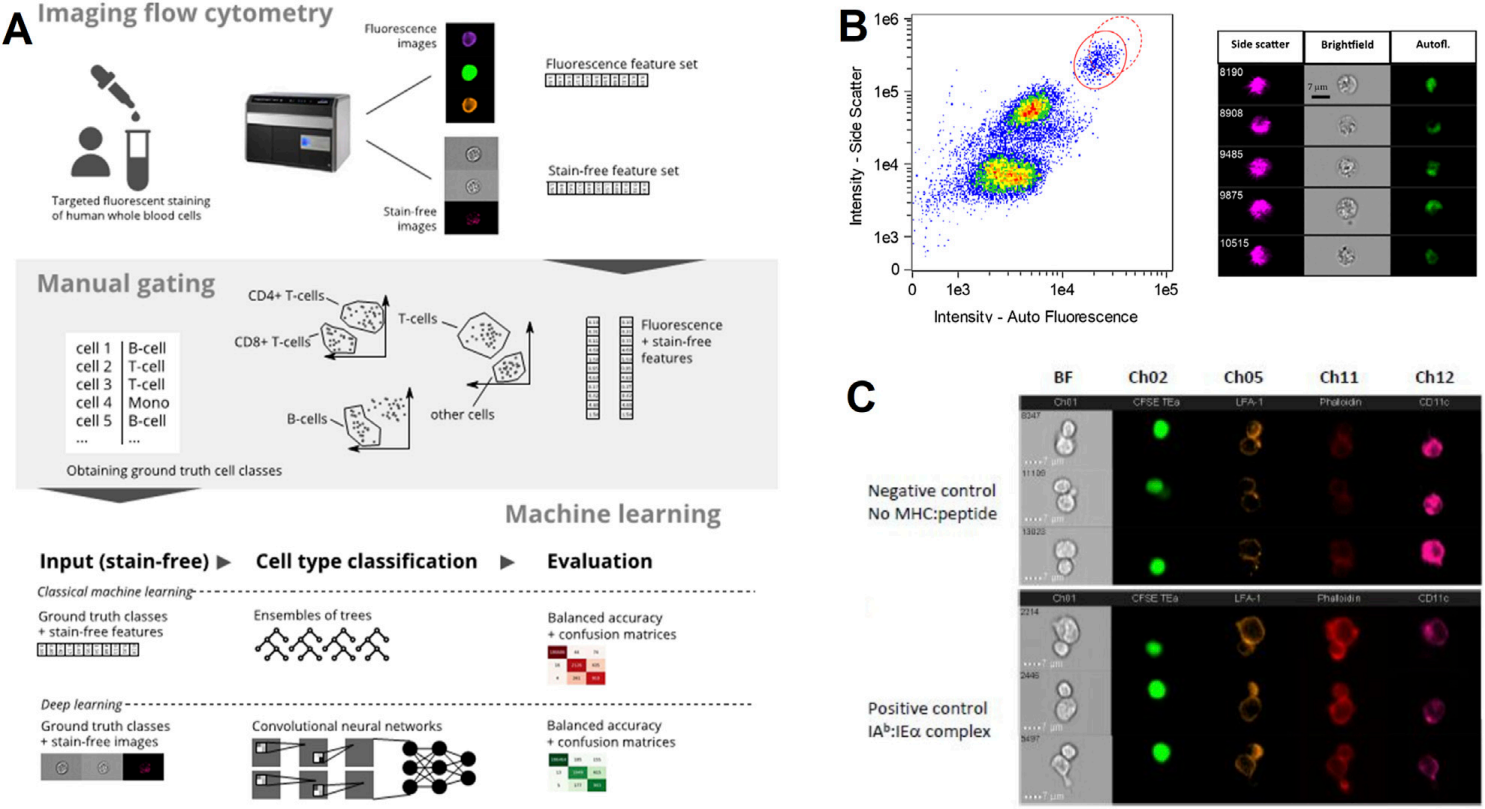
The IFC application in immunology. **(A)** Stain-free leukocyte classification: Machine learning models analyze brightfield and autofluorescence images to classify human white blood cells without fluorescent staining. The workflow includes feature extraction and validation against manually gated populations, achieving high accuracy as reported (Lippeveld et al., 2020). Copyright ^©^ Wiley-VCH. **(B)** Eosinophil activation analysis: Side scatter, brightfield, and autofluorescence images visualize morphological changes in activated eosinophils. Quantitative analysis of cell size, and cytoplasmic texture helps characterize immune cell function (Piasecka et al., 2020). Copyright ^©^ Wiley-VCH. **(C)** Immunological synapse imaging: *Ex vivo* dendritic cells (DCs) and CD4 T cells are labeled for surface adhesion molecules (LFA-1) and intracellular actin (phalloidin). IFC captures dynamic reorganization of the synapse interface, enabling quantification of DC-T cell interactions critical for immune response activation (Markey et al., 2015). Copyright ^©^ Elsevier Ltd.

IFC is instrumental in immunological research, particularly in the analysis of immune cell phenotype and function, showing its unique application value. Conventional FC is based on fluorescence staining for phenotyping immune cells, which has the disadvantages of high staining cost and signal confusion arising from spectral overlap between fluorescent dyes or autofluorescence. Lippeveld, M. et al. used high-quality IFC datasets to evaluate whether machine learning-assisted methods can utilize morphological data from bright and dark field measurements to achieve stain-free classification of a variety of human leukocyte cell types with a balanced accuracy of 74.05% (Figure 3A) (Lippeveld et al., 2020). An advantage of IFC is its ability to capture morphological changes in immune cells during activation, which can be decisive for unraveling the mechanisms of activation and functioning of immune cells, as demonstrated by the quantitative analysis of the activation state of eosinophils using this technique by (Figure 3B) Piasecka et al. (2020). Markey, K. A. and colleagues have devised a technique for evaluating the establishment of immunological synapses between isolated *in vitro* dendritic cells (DCs) and antigen-specific CD4^+^ T cells, utilizing IFC to measure the reorganization of adhesion molecules (LFA-1) and f-actin within DC/T cell interfaces (Figure 3C). This innovative use of IFC marks a significant advancement in research on dendritic cell functions and immune synapses, offering a robust tool for the quantitative, high-throughput examination of DC-T cell interactions both *in vivo* and *ex vivo* (Markey et al., 2015).

### 3.3 Oncology

The application of IFC in the field of oncology focuses on the fine analysis of tumor cell phenotypes, in-depth exploration of the tumor microenvironment, precise resolution of tumor cell heterogeneity, and effective evaluation of tumor treatment response.

**FIGURE 4:**
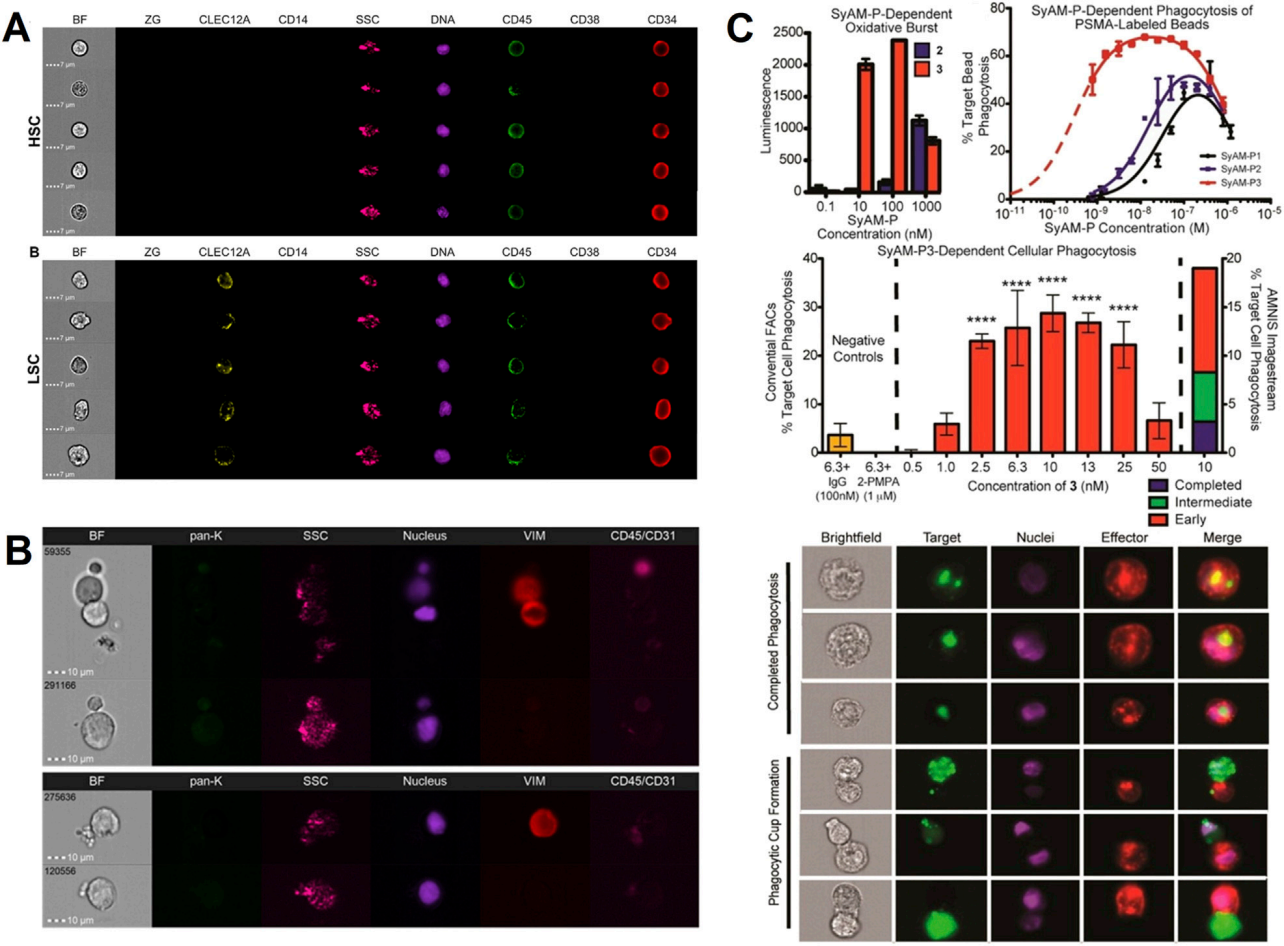
The IFC applications in oncology. **(A)** Leukemic stem cell identification: IFC combined with AI-based image analysis differentiates hematopoietic stem cells (HSCs) from leukemic stem cells (LSCs) in acute myeloid leukemia by morphological features (e.g., nuclear irregularity, surface marker expression) (Hybel et al., 2024). Copyright ^©^ Multidisciplinary Digital Publishing Institute. **(B)** Circulating tumor cell (CTC) characterization: Brightfield and fluorescent images of CTCs from prostate cancer patients highlight morphological heterogeneity. These images aid in understanding tumor metastasis mechanisms (Muchlińska et al., 2022). Copyright ^©^ Multidisciplinary Digital Publishing Institute. **(C)** CAR-T therapy efficacy assessment: SyAM-P molecules guide monocytes to phagocytose prostate cancer cells labeled with PSMA (prostate-specific membrane antigen). IFC visualizes and quantifies phagocytosis efficiency by tracking fluorescently labeled target cells, enabling objective evaluation of immunotherapeutic interactions (McEnaney et al., 2014). Copyright ^©^ American Chemical Society.

Acute myeloid leukemia is a heterogeneous blood cancer with a poor prognosis. It originates from leukemic stem cells arising from the genetic transformation of hematopoietic stem cells. Leukemic stem cells have prognostic value; however, the heterogeneity of their molecular and immunophenotypes poses a challenge for accurate detection due to the lack of specific markers to identify all leukemic stem cells. Hybel, T. E. and other investigators employed IFC in conjunction with AI-assisted image analysis to achieve visual differentiation of leukemic stem cells from hematopoietic stem cells by morphological features, which offers an innovative concept for the advancement of monitoring technologies in acute myeloid leukemia (Figure 4A) (Hybel et al., 2024). Liquid biopsy, as a non-invasive detection method, effectively predicts and monitors the dynamics of tumor development by analyzing metastasis-related substances such as extracellular vesicles, circulating tumor cells and nucleic acids in the patient’s blood. A researcher has used IFC to capture circulating tumor cells and provided typical images of circulating tumor cells for breast cancer and prostate cancer patients, laying a solid foundation for exploring the mechanism of tumor metastasis (Figure 4B) (Muchlińska et al., 2022). In the field of tumor immunotherapy, especially the rapid development of chimeric antigen receptor T-cell (CAR-T) technology has brought significant results for the treatment of a wide range of malignant tumors including leukemias, gliomas, lymphomas, and neuroblastomas (Du et al., 2025; Tang et al., 2023). Patrick et al. from Yale University designed and synthesized SyAM-P mimetic antibody analogs, which were able to simultaneously bind to the PSMA antigen on the surface of prostate cancer cells and FcγRI on the surface of immune cells, effectively guiding immune cells to target prostate cancer cells (Figure 4C). With the help of IFC, the phagocytosis process of immune cells on tumor cells can be clearly observed, and then the efficacy of CAR-T therapy can be intuitively and quantitatively assessed (McEnaney et al., 2014). In addition, by analyzing dipeptidyl peptidase-IV and immunophenotypes by IFC, Rao et al. investigated the mechanism of regulating the dipeptidyl peptidase-IV expression pathway in macrophages, revealing that targeting this pathway may be a novel strategy to attenuate inflammatory responses triggered by dipeptidyl peptidase-IV (Rao et al., 2019). These studies not only provide new technical approaches in the field of oncology, which can not be completed by conventional FC, but also lay the scientific foundation for the implementation of precision medicine.

### 3.4 Microbiology and environmental monitoring

IFC plays a significant role in the field of microbiology and environmental monitoring, particularly in the rapid identification, classification, and quantification of microorganisms, as well as the monitoring of microbial contamination in water and air.

**FIGURE 5:**
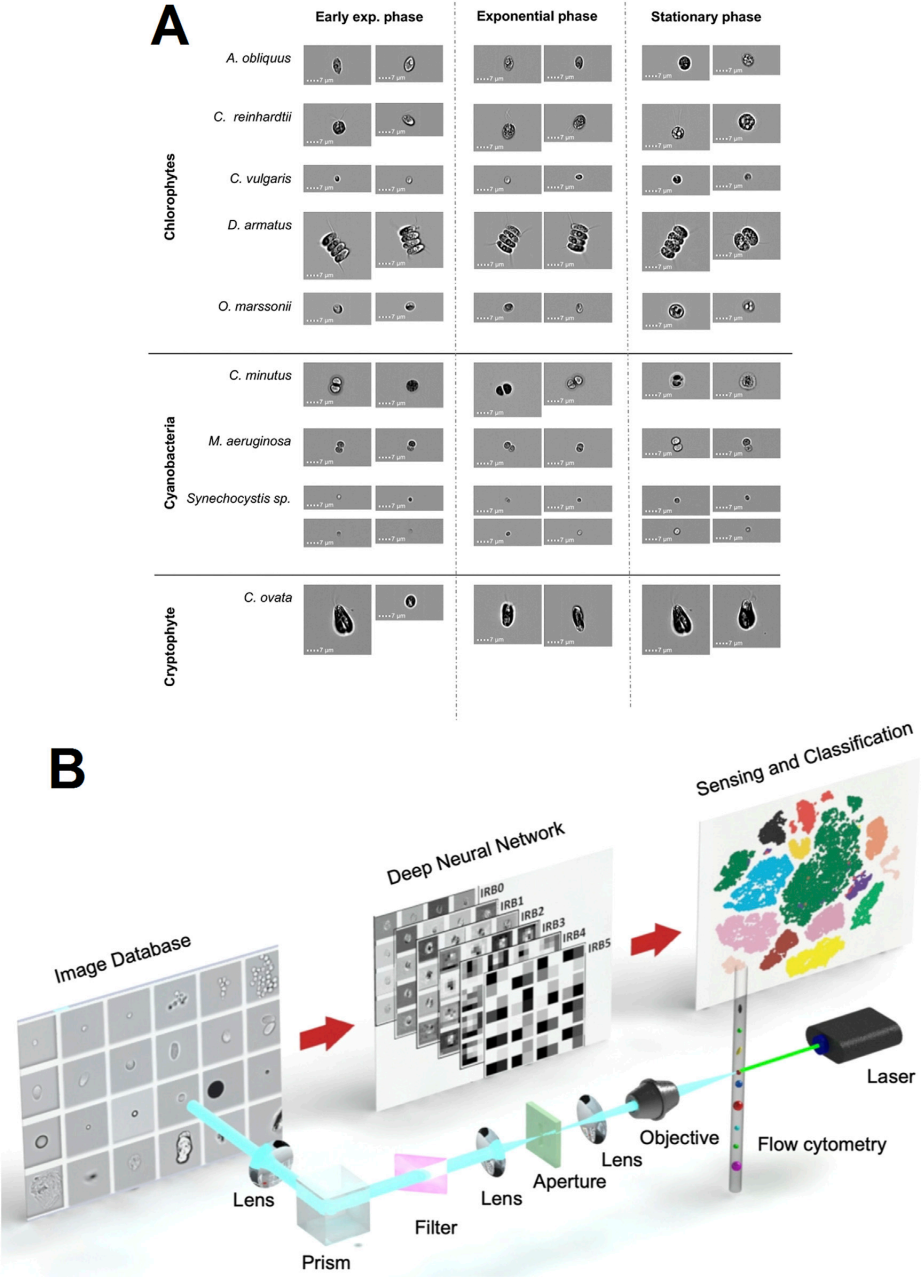
The IFC applications in microbiology. **(A)** Representative brightfield images captured by the IFC of various phytoplankton species utilized in this study for training the deep learning network across three distinct life cycle stages: early exponential, exponential, and stationary phases (Dunker et al., 2018). Copyright ^©^ BioMed Central. **(B)** Introduction to MCellNet, a sophisticated deep learning model designed for IFC to identify Cryptosporidium and Giardia. This integrated setup encompasses a laser source, a flow cytometry module, an imaging apparatus, a repository of images, and a deep neural network (Luo et al., 2021). Copyright ^©^ Wiley-VCH.

In the current context of multiple environmental challenges such as eutrophication, climate warming, and biological invasions, improved identification and enumeration of phytoplankton species have become an integral part of water quality assessment, which is critical for developing effective countermeasures. Using IFC, researcher Dunker S. et al. acquired images of nine common freshwater nano- and micro-phytoplankton species. Based on these images, a deep neural network was trained to successfully recognize phytoplankton species and their life cycle stages, demonstrating an extremely high prediction accuracy of 97% (Figure 5A) (Dunker et al., 2018). In addition, Luo S. and other researchers developed a deep learning-based high-throughput system that combines IFC with an efficient artificial neural network, MCellNet, for the prediction of Cryptosporidium and Giardia flagellates in drinking water, with a classification accuracy of more than 99.6%, and a sensitivity of 97.37% and specificity of 99.95% for the detection of these two pathogens (Figure 5B). This system provides a novel approach for rapid, accurate and high-throughput detection of biological particles for clinical diagnosis, environmental monitoring and potential biosensing applications (Luo et al., 2021).

## 4 IFC data analysis and challenges

### 4.1 Data processing and multi-parameter analysis strategies

The IFC generates high-speed data streams containing rich biological information. The complexity of these data, which often include multiple fluorescent markers, light scattering parameters, and morphological properties, requires efficient data processing strategies and algorithms for processing. For accurate analysis of multicolor-labeled samples, appropriate compensation algorithms are required to eliminate spectral overlap between different fluorescence channels. At the same time, image processing software needs to be able to accurately recognize real signals from background noise to ensure precise identification and tracking of cells or particles. In multiparameter analysis, integrating data from different channels and time points, such as cell size, shape, fluorescence intensity, and other information, is critical for the accurate classification and phenotypic identification of cell populations. In addition to initial analysis using standard tools of conventional FC, such as scatter plots and histograms. A range of new automated data analysis algorithms [t-SNE (van der Maaten and Hinton, 2008), UMAP (McInnes and Melville, 2018), FlowSOM (Van Gassen et al., 2015), PhenoGraph (Levine et al., 2015), etc.] can also be utilized to replace manual data analysis processes. These tasks encompass data preprocessing, the identification and quantification of cell populations, feature extraction, and the classification of samples (Cheung et al., 2021; Mair et al., 2016).

### 4.2 Challenges and solutions for data analysis

The main challenges of high-content data analysis in IFC include the storage, processing, and analysis of large-scale image data. Due to the huge amount of data generated, traditional data processing methods are often unable to cope. One of the strategies to address this challenge is to use high-performance computing platforms and the use of specialized image processing software to significantly shorten the analysis time through parallel processing techniques. At the same time, the development of efficient data compression algorithms and automated image-cleaning processes can also help reduce the burden of data storage and processing. Researchers must craft automated image analysis algorithms to boost the efficiency of data processing, focusing on cell segmentation, feature extraction, and pattern recognition (Hennig et al., 2017). These algorithms, which are usually based on pattern recognition and statistical learning theories, can extract meaningful biological information, such as morphological changes, protein co-localization, and cell-cell interactions, from complex images. Unlike conventional FC, which benefits from standardized, user-friendly analysis tools, IFC’s rich morphological and imaging data often demand tailored machine learning – artificial intelligence approaches. This specificity introduces hurdles such as the need for collaborative expertise between biologists and computer scientists, specialized training in computational methods, and access to high-performance computing resources—all of which are underrecognized limitations in purely biological research contexts.

In addition, While IFC offers unprecedented insights into cellular morphology and high-dimensional phenotypic data, it faces a critical challenge in “data comparability with conventional FC,” stemming from the latter’s established foundational data infrastructure. Conventional FC, with over six decades of technological refinement and widespread adoption, has built a robust ecosystem of standardized protocols, reference datasets, and a vast corpus of comparative literature (De Rosa et al., 2003; Robinson and Roederer, 2015). In contrast, IFC, as a relatively newer technology, lacks the same depth of standardized data frameworks. The key challenges include: 1) Protocol Variability: IFC incorporates morphological features (e.g., nuclear texture, cytoskeletal organization) that are not uniformly defined across studies. For instance, metrics like “nuclear irregularity” or “granule intensity” may be quantified differently based on imaging settings or analysis software, leading to inter-laboratory variability. 2) Limited Reference Datasets: Conventional FC benefits from large-scale repositories (e.g., FlowCAP, Cytobank) hosting millions of standardized datasets, facilitating benchmarking and algorithm validation. IFC, however, has fewer publicly available datasets, particularly for rare cell populations or complex morphological phenotypes, limiting the ability to validate findings against established baselines. 3) Marker Cross-Validation: Many IFC studies rely on fluorescent markers overlapping with conventional FC, but the addition of morphological parameters introduces new variables that lack equivalent historical data. To bridge this gap, data sharing and standardization of analysis processes should be promoted to facilitate collaboration between different laboratories and researchers. 4) High-Throughput Capabilities: In contrast, most commercially available IFCs currently offer lower throughput for multiwell plate processing than conventional FC, mainly due to the trade-off between high-resolution imaging and speed.

### 4.3 Application of AI and machine learning for image analysis

The AI and machine learning techniques provide powerful tools for image analysis in IFC. Deep learning networks can be trained to recognize and classify extremely complex cellular phenotypes, and can even identify subtle differences that are easily overlooked in traditional analysis such as rare cell populations that are lost due to manual gating biases (Mochalova et al., 2022). Machine learning models can be used to predict cellular states, identify abnormal cells, and discover new biomarkers. Algorithms powered by AI can perform image processing and data analysis tasks in seconds to minutes that would otherwise take hours or even days to complete (Lippeveld et al., 2020; Rosenberg et al., 2021). Deep learning models can also assist in removing background noise, enhancing image quality, and performing preprocessing steps such as data normalization, thereby improving the accuracy of the subsequent analysis and significantly improving the efficiency of data processing and analysis in IFC (Doan et al., 2021; Rodrigues et al., 2021). In addition, using predictive models established by deep learning, researchers can predict cellular responses, disease progression, or treatment effects based on previous imaging data (Hybel et al., 2024; McEnaney et al., 2014). These models are able to integrate a large amount of imaging data from different samples and experimental conditions, providing deeper insights for research. In clinical settings, AI decision support systems enhance diagnostic precision. For example, Hybel et al. used IFC with deep learning to identify leukemic stem cells in acute myeloid leukemia, providing clinicians with quantitative tools to guide treatment choices and prognosis assessment (Hybel et al., 2024). In addition, the application of AI technology can facilitate the automation and standardization of IFC, reducing the need for manual manipulation and lowering the variables introduced due to inter-operator variation. Automated workflows not only enhance the reproducibility and reliability of experiments, but also enable non-specialists to perform complex imaging analyses (Mair et al., 2016).

However, there are still some challenges to applying AI and machine learning to IFC analytics. First, large amounts of high-quality and accurately labeled training data are required to optimize algorithms, a process that can be time-consuming and costly. Second, the decision-making mechanisms of machine learning models, particularly deep learning models, are frequently considered “black boxes” due to their lack of transparency, which can be a problem in certain scientific and regulatory environments. In the future, as computing power increases, algorithms improve, and access to large datasets becomes easier, the use of AI and machine learning in IFC will become more widespread. Not only can they improve the accuracy and efficiency of analysis, but they can also help researchers explore unknown biological questions and drive progress in bioscience and medical research.

## 5 Prospects

Technological developments in the field of IFC are advancing at an unprecedented pace. As optical, electronic, and computing technologies continue to advance, we can anticipate qualitative leaps in hardware performance, including higher resolution, faster image acquisition, and lower system costs. For example, new sensor technologies and more efficient photomultiplier tubes will improve detection sensitivity and resolution. In addition, advances in laser technology promise to provide more stable and precise excitation light sources, further optimizing image quality and data accuracy. On the software side, enhanced image processing algorithms, optimized user interfaces, increased automation levels, and enhanced data processing capabilities will make IFC systems more intelligent and easy to use. The integration of real-time data analysis and visualization tools will dramatically improve the user experience and shorten the time from data acquisition to result analysis, which is extremely critical for research and clinical environments that require rapid decision-making, such as the application of intraoperative IFC (D’Amato et al., 2022). In particular, advances in AI and machine learning are pushing IFC forward, enabling researchers to gain a deeper understanding of cellular properties and behaviors, and opening up new possibilities for biomedical research and clinical applications.

With the continuous advancement of IFC technology and its significant improvement in cost-effectiveness, it is increasingly acknowledged for its extensive potential across various emerging fields. In the field of precision medicine, IFC provides a robust scientific foundation for the development of personalized therapeutic plans by accurately analyzing the characteristics of patients’ cells, especially in cancer treatment and immunotherapy (Hybel et al., 2024). In cell therapy, IFC is crucial for assessing the quality and functionality of cell therapy products, such as the phenotypic and functional analysis of CAR-T cells (McEnaney et al., 2014). In drug development, IFC can be used for high-throughput drug screening to assess the effects of drugs on cells, thus accelerating the process of drug discovery and optimization (Rao et al., 2019). In the field of microbiology, IFC can be used for rapid identification and quantification of microbial populations such as bacteria, viruses and algae, providing a powerful tool for environmental monitoring and disease prevention (Luo et al., 2021). In addition, in the field of tissue engineering and regenerative medicine, the technology helps to monitor the behavior and interactions of cells in a three-dimensional culture environment, thereby assessing the efficiency and quality of tissue construction. In the field of food safety, IFC can be utilized for rapid detection of harmful microorganisms and contaminants in food. Finally, IFC has also shown great potential for application in several key areas such as vaccine development, immunization monitoring and cancer screening (Lippeveld et al., 2020).

Future research will likely focus on innovative applications at the intersection of multiple disciplines, particularly in the convergence of biology, materials science, and computer engineering to drive the development of novel IFC systems. In addition, with the further development of big data and AI, it is expected to see more research on how machine learning can be utilized for image recognition and classification to significantly increase the level of automation and accuracy of the analysis process. Another research focus is likely to be on improving the versatility and flexibility of the system so that it can perform multiple tasks at the same time, such as acquiring morphological, genomic, and proteomic data from the same sample at the same time. This will not only help provide more comprehensive biological information, but will also facilitate the scope of application of the system in more complex and dynamic biological processes.

## 6 Conclusion

As a cutting-edge biotechnology that combines the high-throughput analytical advantages of FC with the high resolution of imaging technology, IFC greatly expands the ability of researchers to characterize cellular properties in higher dimensions and with greater precision. The core value of this technology is that it provides detailed visual information on cell morphology, size, fluorescent markers, and internal structure, providing valuable data for an in-depth understanding of cellular functions, disease mechanisms, and the development of innovative therapies. In the field of biomedical research, IFC exhibits a wide range of applications, including but not limited to the monitoring and classification of cell types in immunological studies, and the identification and quantification of rare cells, such as circulating tumor cells, in cancer research, which is critical for early diagnosis and treatment. In addition, the technology also plays a key role in drug discovery and toxicology evaluation, accelerating the screening and optimization of new therapeutic strategies by assessing the efficacy and safety of drug candidates.

In the future, along with the deep integration of AI and deep learning technologies, IFC’s analytical performance is expected to realize a qualitative leap. Automated image analysis technology will significantly shorten the data processing time, while improving the accuracy and objectivity of the analysis. At the same time, technological advances will promote the popularization of IFC, making it a powerful tool for more research laboratories and medical institutions to carry out cutting-edge research. In conclusion, IFC is of great significance to the advancement of biomedical science and will continue to play a key role in the development of precision medicine, disease diagnosis and treatment strategies that will revolutionize future medical diagnosis and treatment.
